# On Iron Metabolism and Its Regulation

**DOI:** 10.3390/ijms22094591

**Published:** 2021-04-27

**Authors:** Anne-Cathrine S. Vogt, Tasneem Arsiwala, Mona Mohsen, Monique Vogel, Vania Manolova, Martin F. Bachmann

**Affiliations:** 1 Department of Rheumatology, Immunology and Allergology, Inselspital, University Hospital Bern, 3010 Bern, Switzerland; anne-cathrine.vogt@students.unibe.ch (A.-C.S.V.); tasneem.arsiwala@gmail.com (T.A.); mona.mohsen@dbmr.unibe.ch (M.M.); monique.vogel@dbmr.unibe.ch (M.V.); 2 National Centre for Cancer Care & Research (NCCCR), Doha 3050, Qatar; 3 Vifor (International) AG, 9001 St. Gallen, Switzerland; Vania.Manolova@viforpharma.com; 4 Nuffield Department of Medicine, Jenner Institute, University of Oxford, Oxford OX3 7BN, UK

**Keywords:** iron, macrophages, hepcidin

## Abstract

Iron is a critical metal for several vital biological processes. Most of the body’s iron is bound to hemoglobin in erythrocytes. Iron from senescent red blood cells is recycled by macrophages in the spleen, liver and bone marrow. Dietary iron is taken up by the divalent metal transporter 1 (DMT1) in enterocytes and transported to portal blood via ferroportin (FPN), where it is bound to transferrin and taken up by hepatocytes, macrophages and bone marrow cells via transferrin receptor 1 (TfR1). While most of the physiologically active iron is bound hemoglobin, the major storage of most iron occurs in the liver in a ferritin-bound fashion. In response to an increased iron load, hepatocytes secrete the peptide hormone hepcidin, which binds to and induces internalization and degradation of the iron transporter FPN, thus controlling the amount of iron released from the cells into the blood. This review summarizes the key mechanisms and players involved in cellular and systemic iron regulation.

## 1. Introduction

Iron (Fe) is one of the most abundant elements of the Earth’s crust [1]. As a transition metal, its ability to donate and accept electrons in redox reactions, makes it favorable for fundamental biological processes [2]. Therefore, iron is one of the most important metals to sustain life from single cell bacteria to multi-cellular organisms such as humans [3,4]. This metal plays a vital role in several cellular processes such as DNA synthesis, nucleic acid repair, cellular respiration in mitochondria, cell growth and cell death and contributes to host defense and cell signaling [1,2]. On top of these diverse roles, iron incorporated into heme is the main component of hemoglobin (Hb) and is thus crucial for oxygen transport and supply by erythrocytes [1]. With the capability of donating and accepting electrons, metal can be found in two oxidation states in the human body [1]. Although iron is essential for the functioning of human physiology, iron also has the potential to be toxic in the presence of hydrogen peroxide (H_2_O_2_) [5]. Divalent ferrous iron (Fe^2+^) is a cation capable of reacting with hydrogen peroxide generating one of the reactive oxygen species (ROS), the hydroxyl radical while being oxidized to Fe^3+^ [6]. The radicals generated in these so-called Fenton or Fenton-like reactions can cause oxidative damage and induce lipid peroxidation and tissue injury [6,7]. The hydroxyl radical is known as one of the most dominant oxidants found in the human body attacking proteins, lipids, nucleic acids and carbohydrates leading to peroxidation and cell apoptosis [3]. As iron is harmful when present at high concentrations, tight regulation is required to avoid iron overload [8]. Additionally, prolonged iron deficiency leading to reduced iron availability, causes iron-restriction erythropoiesis in the bone marrow resulting, in moderate to severe anemia [9,10]. Furthermore, persistent iron deficiency even without anemia has been associated with fatigue, poorer cognitive and motor skills, defective immune cell function and increased disease severity in heart failure [11].

## 2. Iron Flow in the Human Body

**Figure 1:**
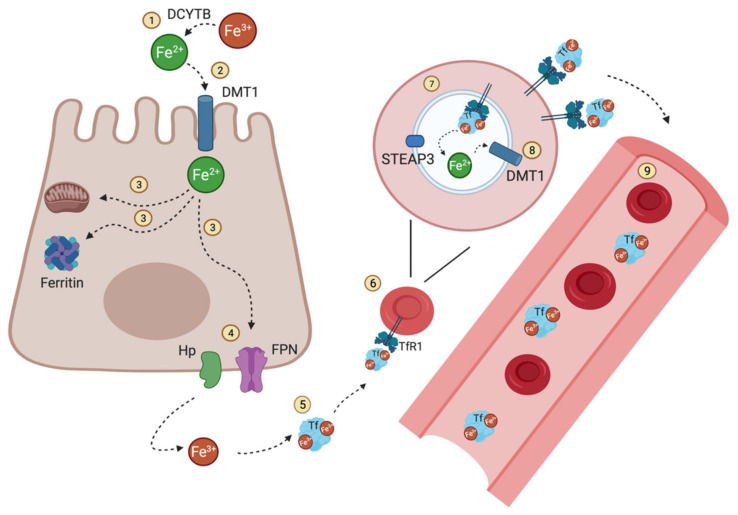
**Iron distribution and circulation.** Nonheme dietary iron Fe^3+^ is reduced to Fe^2+^ by the iron reducing DCYTB (**1**) prior to its uptake at the apical membrane of enterocytes via DMT1(**2**). Fe^2+^ can then be directly used for intracellular mechanisms, stored when bound to ferritin or released directly into the circulation (**3**). (**4**) Therefore, reduced iron Fe^2+^ is transported by ferroportin (FPN), the only known iron exporter so far, and then oxidized by hephaestin Hp to be then bound to Tf (**5**). Most of the iron present in the circulation is bound to Tf. As a result, erythrocyte precursors (erythroblasts) take up this transferrin-bound iron via TfR1(**6**). Fe^3+^ bound to transferrin is reduced in the endosome by ferrireductase STEAP3 to Fe^2+^ (**7**) where it is exported via DMT1 (**8**) into the cytosol and enters the labile iron pool. Mature RBCs circulate in the blood for around 120 days (**9**) until they are removed from the circulation during erythrophagocytosis. The illustration was created using BioRender.com, (accessed on 3 April 2021).

Most of the iron in the human body is associated with erythrocyte hemoglobin (~80%). The rest is stored in macrophages and hepatocytes or active in other heme-groups or Fe-S clusters [12]. Most of this iron is required for erythropoiesis, which is the production of oxygen-transporting red blood cells [13,14]. An erythrocyte might contain around 280 million molecules of hemoglobin, resulting in an iron quantity of over 1 billion atoms per red blood cell [15]. Hemoglobin’s primary function is oxygen transport and delivery to tissues [15]. As the circulating iron pool is comparably small compared to the daily iron demand, iron has to be continuously recycled from old red blood cells to reach the daily requirement of iron to maintain erythropoiesis and other bodily needs [2]. A comparably low amount, only 1-2mg of iron, is provided by dietary absorption [2]. Dietary iron occurs in two forms, heme and nonheme bound iron [16]. Major sources of heme iron are hemoglobin and myoglobin contained in meat and poultry, whereas non-heme iron sources are cereals and vegetables [16]. Thus, due to extensive recycling, only 1–2 mg of absorbed iron per day is required to counterbalance losses from shedding cells to maintain homeostatic balance [17]. Before its uptake, dietary non-heme iron (Fe^3+^) must be reduced to Fe^2+^ (ferrous iron) for intestinal uptake [2]. Ferric iron (Fe^3+^) is thought to be reduced to Fe^2+^ by the iron reducing ferric reductase duodenal cytochrome B (DCYTB) at the apical membrane of enterocytes, facing the gut lumen [3]. This reduced iron enters the body through the apical membrane of enterocytes via divalent metal transporter 1 (DMT1, also known as Nramp2), the major regulatory unit for iron absorption in the duodenum and upper ilium in the small intestine [1,18]. Iron taken up by enterocytes can directly be used for intrinsic cellular metabolic mechanisms, stored in ferritin or released across the basolateral membrane for systemic iron delivery [2]. After absorption in enterocytes, reduced iron Fe^2+^ is transported to the circulation through ferroportin (FPN1), the only iron efflux protein, and after oxidation by hephaestin (Hp) or ceruloplasmin to Fe^3+^ it binds to the main plasma iron carrier transferrin (Tf) for further use [2,19] (Figure 1). Transferrin (Tf) is composed of two high-affinity binding sites for iron Fe^3+^ and acts as the major transport glycoprotein for ferric iron and maintains iron in its inert state [2,20]. Under normal conditions, most of the iron circulating in the blood is bound to transferrin. In the case of certain pathological states, when the iron binding capacity of Tf is exceeded, non-transferrin bound iron (NTBI) can occur, which may be taken up by the liver with toxic consequences [21]. One redox-active component of NTBI is the so-called labile plasma iron (LPI), which is unavailable for erythropoiesis. LPI can be taken up by non-hematopoietic cells causing parenchymal iron deposition which can result in free radical damage [22].

Most of the iron present in the blood is bound to transferrin. Erythrocyte precursors in the bone marrow are restricted to take up transferrin-bound iron via transferrin receptor 1 (TfR1), as they express high levels of TfR1, whereas hepatocytes and other non-erythroid cells are also able to use NTBI and other sources of iron as they express other transporters [23]. Circulating iron delivered to erythroid precursors and other cells is taken up via receptor-mediated endocytosis into clathrin-coated pits [2]. TfR1 is a receptor protein with a size of 97-kDa, harboring a high-affinity receptor for Tf carrying two Fe^3+^ (diferric Tf), and is composed of a homodimeric protein stabilized by disulfide bonds [21,24]. Diferric Tf is bound to TfR1 and then internalized by clathrin-dependent endocytosis [25]. Binding of diferric Tf to TfR1 is pH-dependent [24]. Tf bound iron (Tf-Fe_2_) is released on acidification of the endosome [17]. Iron, following its reduction to Fe^2+^ by STEAP3, is then exported to the cytosol via DMT1, and TfR1 is recycled back to the cell surface [2,26]. At this point, iron enters the labile iron pool and is either utilized directly and incorporated into heme or stored in cytosolic ferritin [2]. Interestingly, intracellular iron is generally present in the form of Fe^2+^, while extracellular iron is Fe^3+^. This probably reflects and maintains cellular physiological integrity with the cytosol having more reducing properties than the extracellular environment.

Ferritin (Ft) is a protein composed of 24 subunits containing heavy chains (H) and light chains (L) [20]. The assembly of these subunits allows the protein to bind up to 4500 atoms of iron and therefore makes it the major iron storage protein in cells [26]. The subunits build a cage-shaped complex binding and storing Fe^3+^ ions in their inert form that restricts the generation of damaging redox reactive species [2]. Ferritin-bound iron is the major mechanism of iron storage in macrophages and liver hepatic cells. Other cell types, such as erythroblasts are able to take up ferritin-bound iron and utilize iron to support their differentiation [19,20]. Iron released from Ft is regulated by a process known as ferritinophagy, where the nuclear receptor coactivator 4 (NCOA4) directly binds the Ft light chain and transfers the complex to the autolysosome for degradation [27]. During the process iron is released and becomes available for biosynthetic pathways [27]. 

## 3. Liver the Central Organ in Iron Homeostasis

The liver, especially hepatocytes, play a major role in iron metabolism. Hepatocytes are the most common cell type (around 80% of liver mass) in the liver and hepatocytes also contribute most to overall iron metabolism. Hepatocytes are able to synthesize a high amount of the iron storage protein ferritin (Ft). Due to this fact, hepatocytes act as major storage localization for absorbed iron [21]. The liver also produces most of the transferrin, located in the plasma for binding absorbed iron [21]. In the blood, most of the iron present is bound to transferrin, so-called transferrin-bound iron (TBI), but a small pool is also present as NTBI [28]. NTBI is likely to be the major contributor to iron loading of hepatocytes when transferrin is saturated [17]. In addition, liver hepatocytes act as the central regulators of iron homeostasis by producing and releasing the 25 amino-acid peptide hormone hepcidin [28]. Hepcidin is secreted into the bloodstream and inhibits the release of iron in several cells, such as duodenal enterocytes, macrophages, hepatocytes and Kupffer cells [21]. By binding to FPN, the only known cellular iron export protein, hepcidin mediates FPN’s ubiquitination, internalization and degradation as well as directly blocking the channel, resulting in the blockade of iron export out of the cell into the plasma [29]. The synthesis of the peptide hormone is regulated at the transcriptional level controlled by serum iron concentrations [10]. When serum iron levels are increased, hepcidin expression is upregulated and results in the blocking of iron transport to plasma via FPN, thus providing a negative feedback response preventing potential toxic iron accumulation in the body [1,10]. Decreased iron plasma levels result in low transferrin saturation [16], causing reduced synthesis of hepcidin [10]. Therefore, iron concentration in biological fluids is tightly controlled to provide adequate intracellular and extracellular iron levels and prevent its toxic accumulation [16]. This is a key process, as any abnormalities in the distribution and content of iron in the body can have harmful effects on the physiological processes [30].

## 4. Iron Regulation

### 4.1. Cellular Regulation of Iron—The Iron Regulating Proteins (IRPs)

Iron metabolism is regulated at both the systemic and cellular levels [31]. In all vertebrates, the major protein involved in iron transport is TfR1 [32]. Differic-transferrin is taken up via the transferrin receptor. The Tf/TfR1 complexes are endocytosed through the clathrin-dependent pathway [33]. Acidification within the endosome leads to a conformational change in Tf and TfR1, resulting in the dissociation of iron from Tf [33]. After dissociation, the Tf-Tf1R complex is recycled back to the plasma membrane [24]. The homologous protein of TfR1, transferrin receptor 2 (TfR2), is ubiquitously expressed in hepatocytes [33,34]. At the cellular level, the regulation of the expression of proteins involved in iron metabolism and homeostasis, such as ferritin or the transferrin receptors, is coordinated through the interaction of iron sensing proteins, known as iron regulatory proteins (IRPs) or IRE-binding proteins, where IRE stands for iron-responsive elements [32]. The IRE/IRP regulatory system was first described in the late 1980s [31]. This network controls iron homeostasis by regulating gene expression post-transcriptionally [35]. IREs are present in mRNA and are extremely conserved IRP binding sites, with a hairpin structure of 25–30 nucleotides, which are found in the untranslated region (UTR) of the mRNA transcripts encoding the H and L subunits of ferritin, in TFR1 as well as in several other genes related to iron metabolism [36]. IRP1 and IRP2 are homologous to the aconitase gene family, possessing the ability to sense cytosolic iron concentration and modify gene expression of their mRNA targets correspondingly. Thus, the IRP/IRE system is a key component for the organism, enabling the cells to rapidly accommodate cytosolic iron and facilitate the functioning of numerous iron-dependent cellular components at the post-transcriptional level [36].

### 4.2. Sensing and Regulating Intracellular Iron by IRP1 and IRP2

**Figure 2:**
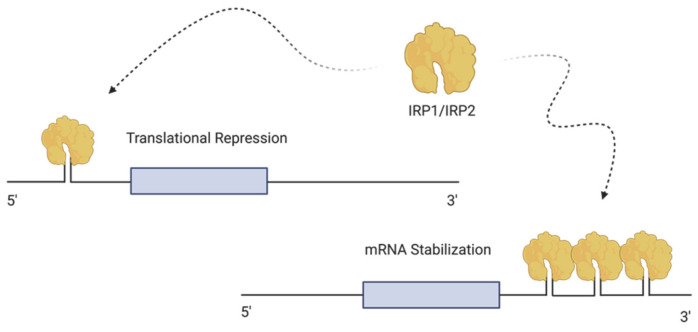
**Cellular iron regulation.** The intracellular iron pool is regulated by the binding of IRP1 and IRP2 to IRE. IRP1 and IRP2 are RNA binding proteins that interact with IRE to control the translation of proteins involved in iron metabolism. IRPs are either present at the 3′ UTR or the 5′ UTR of the target mRNA. When an IRP binds to a single IRE at the 5′ UTR, mRNA translation is repressed. On the other hand, the binding of IRP to IRE at the 3′ UTR stabilizes the transcript and leads to increased mRNA translation. The illustration was created using BioRender.com, (accessed on 3 April 2021).

The intracellular iron pool regulates the binding of IRP1 and IRP2 to the IRE [33]. IRP1 (90kDa) and IRP2 (105kDa) are RNA-binding proteins that interact with IRE to control the translation of ferritin and FPN mRNA and additionally control the stability of TfR mRNA [37] (Figure 2). High iron levels, leading to the assembly of cubane [4Fe-4S] clusters in IRP1, promote the inhibition of IRP1 binding activity to IRE, thereby leading to the conversion of IRP1 to aconitase [33]. In iron starved cells, each IRP binds with high affinity to IREs [36]. The translation of iron-related proteins is dependent on the location of the IRE on the UTR. IREs can be present either at 3′ UTR or 5′ UTR of the target mRNA [37]. IRP prevents the translation of mRNA when binding to a single IRE located in the 5′ UTR region, whereas the binding of IRP to IREs at the 3′ UTR protects the transcript from endonucleolytic cleavage and degradation [36]. For example, under low iron conditions, IRPs bind to IREs of the 5′ UTR of FPN and ferritin mRNAs mediating their degradation, leading to a decrease in iron storage and export. However, simultaneously, the binding of the IRP’s at the 3′ UTR of TfR1 and DMT1 mRNAs will stabilize the transcripts, leading to increased iron import [1]. In contrast, in iron-loaded cells, IRPs will not bind to the IREs located at the 5′ UTR of transcripts, leading to their continuous translation. Conversely, transcripts possessing IREs at the 3′ UTR will undergo endonuclease cleavage, leading to the subsequent degradation of the cleavage products [36].

### 4.3. Systemic Regulation of Iron—The Hepcidin–Ferroportin Axis

At the systemic level, iron homeostasis is regulated via the hepcidin-/ferroportin (FPN) axis [38]. Hepcidin acts as a negative regulator of iron flows [22]. FPN expression plays a crucial role in controlling iron release from enterocytes, liver hepatocytes and macrophages [39]. FPN is composed of 12 transmembrane helices divided into two halves forming two lobes [14]. The lobes can change between two conformational states. In the active first state, the central cavity is facing the intracellular space and is therefore not accessible from the outside. In the second conformational state, the cavity is open to the extracellular space and therefore no longer accessible from the intracellular side [14]. In steady state conditions, iron is released from the main iron stores through FPN [1]. For controlling plasma and tissue iron levels, FPN expression is tightly regulated at the posttranslational level by circulating hepcidin [18]. Dysregulation of hepcidin expression results in iron disorders [16]. Hepcidin deficiency induces iron overload in hepatocytes such as in hereditary hemochromatosis [40]. In contrast an overproduction of hepcidin is associated with iron-restricted anemia [41]. Hepcidin is only regulated at the transcriptional level, mainly expressed in liver hepatocytes. The peptide undergoes proteolytic processing, leading to a bioactive molecule released into the bloodstream and there being able to bind and block FPN activity [1,42]. Besides hepatocytes, monocytes, macrophages and the kidney are also able to produce hepcidin but to a lower extent [43]. Binding of hepcidin to FPN expressed on macrophages, hepatocytes and other cell types induce internalization and lysosomal degradation of the iron exporter [18]. Thus, iron export is blocked in FPN-expressing cells, leading to cellular retention of iron [1]. For example, in a situation of iron overload, hepcidin expression is increased, and iron-export through FPN is blocked. Thus, when a high level of iron in the circulation causes increased cellular uptake of iron, hepcidin expression is enhanced to prevent cellular export of iron to avoid systemic iron accumulation in the extracellular space [1]. Hepcidin expression is inhibited under anemia or ineffective erythropoiesis but stimulated under a high iron load and inflammation [42]. Additionally, it has been shown that ERFE is an important regulator of hepcidin expression and is needed for the rapid response to acute hemorrhage [44].

### 4.4. Regulation of Hepcidin through the Bone Morphogenetic Protein

**Figure 3:**
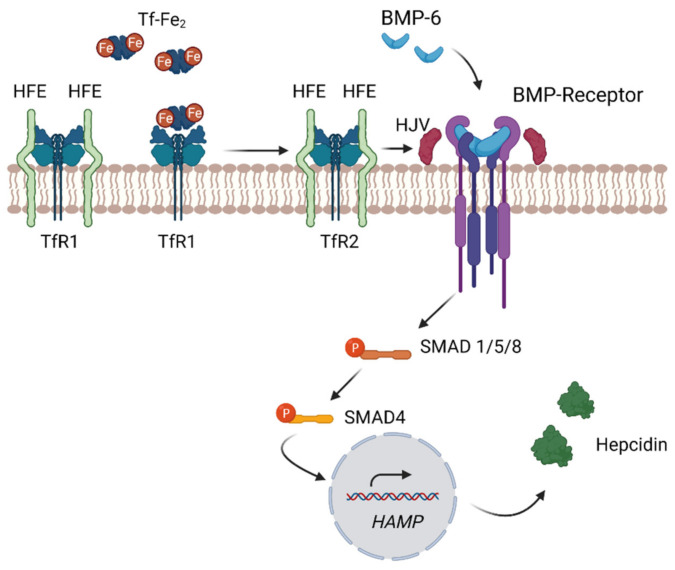
**Regulation of Hepcidin Expression.** Circulating hepcidin regulates the amount of iron released into the blood from macrophages and especially hepatocytes. Decreased hepcidin expression occurs when the rate of erythropoiesis increases (e.g., in response to anemia), leading to increased ferroportin expression causing increased iron transfer into blood. In contrast, hepcidin expression is increased by elevated plasma iron (Tf-Fe_2_) or inflammation to counteract an oversaturation of Tf or iron loss, preventing the formation of cytotoxic NTBI. The liver directly senses circulating iron bound to Tf or indirectly in response to iron-induced BMP6. Increased hepatic iron levels induce the expression of BMP6. BMP6 stimulates hepcidin expression by binding to the BMP receptor and HJV, leading to intracellular signaling via SMAD proteins, coupled with SMAD4 translocating to the nucleus and inducing hepcidin expression. The liver directly senses circulating iron by expression of TfR1 and TfR2. With increasing serum iron levels, Tf-Fe_2_ binds to TfR1 and HFE binding to TfR2 is induced. This complex interacts with HJV and enhances the BMP signaling pathway, leading to hepcidin transcription. The illustration was created using BioRender.com, (accessed on 22 April 2021).

Hepcidin expression is principally regulated transcriptionally by iron in a feedback loop involving multiple pathways by which hepatocytes recognize circulating iron status [22]. The regulation of hepcidin synthesis is complex and involves several proteins present at the plasma membrane of hepatocytes, i.e., hereditary hemochromatosis proteins (HFE) and transferrin receptor 2 (TfR2) as well as hemojuvulin (HJV) [10]. They tightly regulate the expression level of hepcidin by the bone morphogenetic protein 6 (BMP-6) [10]. BMP-6 is an extracellular signaling molecule, belonging to the transformation growth factor (TGF-β) superfamily, expressed in hepatocytes [17,42]. Elevated intracellular liver iron concentration enhances the expression of BMP6 [11]. Binding of BMP-6 to its corresponding BMP receptor (BMPR) and hemojuvelin (HJV), a BMP co-receptor, on hepatocytes, initiates intracellular signaling transduction via SMAD proteins [11,18]. This pathway involves phosphorylation of SMAD1, 5 and 8 (pSMADs) accompanied with pSMADs/SMAD4 translocating to the nucleus [23]. This then activates the transcription expression of the hepcidin-encoding gene (HAMP) [11,18]. Serum iron levels may activate hepcidin expression autonomously of BMP6 [11]. Hepatocyte transferrin receptor 1 and 2 (TfR1, TfR2) and HFE function as extracellular iron sensors and specifically sense circulating concentrations of transferrin-bound iron [41]. As HFE competes with Tf for binding to TfR1, HFE associates with TfR1 when serum iron levels are low and is displaced when the receptor binds Tf-Fe_2_ [45]. With increasing serum iron concentrations, Tf-Fe_2_ binds TfR1, leading to the displacement of HFE [11]. The released HFE then interacts with TfR2 [11]. This HFE/TfR2 complex then interacts with hemojuvelin (HJV) and induces the BMP signaling pathway [22] leading to hepcidin production (Figure 3). Similarly, to Hepcidin-deficiency, HFE deficiency causes hereditary hemochromatosis.

As a consequence, loss of function of hemojuvelin, BMP6 and SMAD4 in knock-out mice leads to low hepcidin levels, resulting in iron overload, demonstrating the master regulatory role of hepcidin in iron homeostasis [46]. Additionally, BMP-6 seems to be one of the major ligands responsible for the activation of hepcidin expression, as BMP-6 knockout mice showed a severe iron overload due to the failure to activate hepcidin expression [43].

### 4.5. Hepcidin Regulation by Inflammation

**Figure 4:**
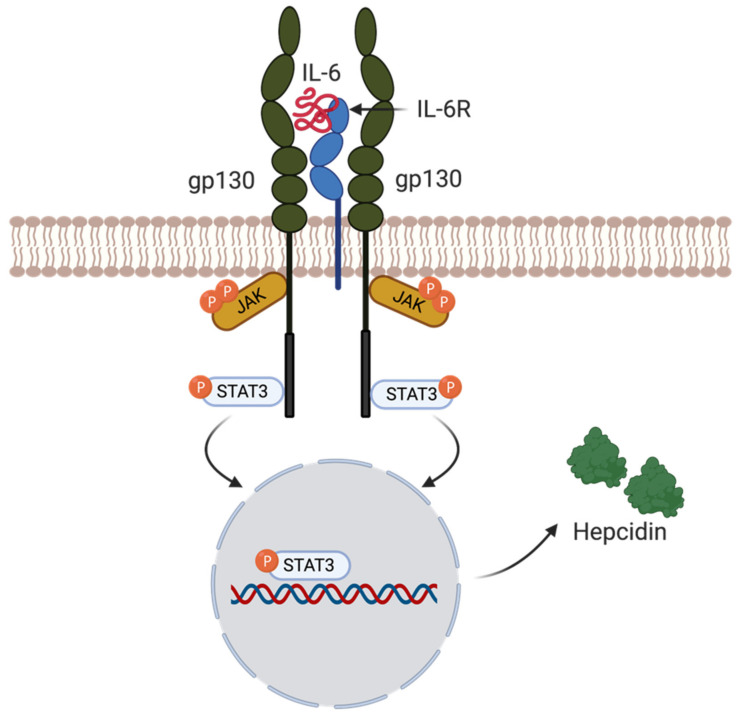
**Inflammation triggering hepcidin expression.** The inflammatory cytokine interleukin 6 (IL-6) can lead to hepcidin induction via the IL-6R/STAT3 pathway. The binding of IL-6 to its corresponding receptor IL-6R leads to the downstream phosphorylation of STAT3 via JAK1/2. After its phosphorylation, STAT3 will translocate into the nucleus binding to the hepcidin promotor inducing hepcidin expression. The illustration was created using BioRender.com, (accessed on 3 April 2021).

Inflammatory cytokine interleukin-6 (IL-6) can also trigger hepcidin induction via the IL-6R/STAT3 pathway [38]. Inflammatory cytokines generated in the context of infections with iron-dependent invading pathogens, stimulate an innate immune response [42]. This pathway is mediated by macrophages releasing IL-6 during infection and inflammation, leading to increased hepcidin levels, mediated by STAT3 signaling, resulting in iron sequestration [39]. In more detail, IL-6 binds to the gp130 protein receptor complex mediating a JAK1/2 tyrosine-kinase-mediated phosphorylation of the transcription factor STAT3. When STAT3 is activated, it is translocated into the nucleus and binds to the STAT3-responsive element on the hepcidin promoter, leading to the induction of hepcidin transcription [42] (Figure 4). STAT3 signaling therefore is an additional pathway to SMAD for stimulation of hepcidin production. Stimulation of hepcidin expression during an infection, induced via IL-6, greatly decreases the access of bioavailable iron to invading pathogens [42]. Therefore, hepcidin expression and FPN degradation play a significant role in iron homeostasis and immunity. As a consequence of low extracellular iron levels due to hepcidin, pathogens, such as *Yersinia pestis*, the causative agent of the plague, produce potent iron chelators (siderophors) to overcome the scarcity of the metal (see also below) [47].

## 5. Macrophages in Control of Iron Homeostasis

Macrophages play an essential role in maintaining and regulating iron homeostasis, which was already proposed late in the 19th century by Metchnikoff [48]. Based on their origin, tissue-resident macrophages can be divided into two subgroups. The first group has its origin in the yolk sac and is maintained by self-renewal and proliferation, whereas the second subset evolves from hematopoietic precursors and circulating monocytes [49]. Tissue-resident macrophages assist bystander parenchyma cells in their function and contribute to tissue repair and regeneration [48]. Besides their central inevitable role in immunity, macrophages play a central role in iron homeostasis by regulating and controlling cellular iron import and export [1,48]. Tissue macrophages take up iron via several receptors, such as transferrin receptor protein (TfR1), LDL-related receptor (CD91) and CD163 (the hemoglobin-haptoglobin receptor), which bind to Tf-bound iron, heme-hemopexin (Hx-heme) and hemoglobin-haptoglobin (Hb-Hp), respectively [50]. After the reduction of Fe^3+^ to ferrous iron Fe^2+^ within the endosomal compartment, Fe^2+^ enters the cytosolic labile iron pool (LIP) via the transporter DMT1 [51]. The intracellular labile iron pool is used for iron storage, export and trafficking. A majority of the ferrous iron is transported to the mitochondria and incorporated into heme or Fe–S clusters to assist in the electron transport chain for energy production [52]. The leftover ferrous iron within the LIP that is not metabolized or exported is then further stored in the cytosol bound to ferritin, a nontoxic heteropolymer caging the excessive iron [53]. On the cellular level, iron is needed for cell growth, repair and even some forms of cell death [54], so called ferroptosis [30]. Ferroptosis is a relatively new type of programmed cell death. Although already described as a phenomenon in 2003, the biological concept of this process was first proposed in 2012 by Dixon. This type of cell death is iron dependent and characterized by the accumulation of lipid ROS [30].

On the systemic level, iron is redistributed to support erythropoiesis and the production of many enzymes involved in redox-functions [50].

### 5.1. Macrophage and Erythropoiesis

Erythropoiesis is the process where mature red blood cells from multipotent stem cells are generated [55]. This RBC production is the single largest consumer of iron in the body [54]. RBCs perform one of the most critical functions in the human body, transporting oxygen to all organs and tissues, where oxygen binds to the iron atoms in the heme part of hemoglobin [56]. Bone marrow steady state erythropoiesis is a homeostatic process, where new erythrocytes are produced at a constant rate to replace senescent red blood cells [57]. This process can be divided into two parts, the first phase is the proliferation and maturation of progenitors, which are erythropoietin (EPO) dependent [55]. EPO induces the production of erythroferrone (ERFE), produced by erythroblasts, which acts on hepatocytes to suppress the production of hepcidin, stimulating iron absorption as well as the release of iron from red blood cell recycling macrophages [58]. The second phase includes the differentiation from proerythroblasts to red blood cells [55]. This second step is strongly iron dependent, where the requirement of iron is involved in metabolically active and dividing cells. Independent of hemoglobin, the metal acts as an important cofactor for enzymes vital for actively dividing cells, including the hematopoietic stem cells during erythropoiesis [54]. Considering that proerythroblasts are rapidly proliferating cells, they thus requiring a constant supply of iron contributed by Tf [59]. Since iron is required in large amounts for hemoglobin synthesis in maturing erythroblasts, approximately 25 mg of iron must be supplied to the bone marrow for the daily production of erythrocytes. Most of this iron is provided by macrophages, recycling the metal from old RBCs [55]. The macrophage lineage with the capacity of erythrophagocytosis originates essentially from bone marrow derived monocyte progenitors [60]. These macrophages phagocytose senescent erythroblasts and extract the contained iron from these cells and recycle it for further use [24]. As NCOA4 contributes to the regulation of cell and systemic iron homeostasis, Nai et al. pointed out that NCOA4 has a crucial function for ferritinophagy in macrophages to sustain erythropoiesis [61].

### 5.2. Red Pulp Macrophages in the Spleen

The spleen is the primary organ to filter out senescent red blood cells from the system [62]. The spleen has two parts; one known as the red pulp is in charge of filtering the circulating blood and the second part, the white pulp, is committed to adaptive immunity [62]. These two parts are divided by the marginal zone [63]. Tissue-resident macrophages in the red pulp of the spleen, so-called red pulp macrophages (RPM), recognize aging RBCs, which are then taken up by erythrophagocytosis [1]. RPMs mediate the turnover of billions of senescent erythrocytes per day [64]. Upon aging, the plasma membrane of RBCs undergoes destructive changes, which makes them susceptible to be recognized and engulfed by macrophages [62]. Senescent RBCs exhibit reduced expression of CD47 on their surface, a molecule that acts as a prominent “do not eat me” signal. This reduction in CD47 expression on the surface of senescent RBCs is thought to enable the elimination of aged RBCs by macrophages [19]. Besides the reduced expression of CD47 on the cell surface other removal signals such as phosphatidyl exposure, the oxidation of proteins and lipids as well as the activation of adhesion molecules may contribute to the sequestration and final erythrophagocytosis [64] The plasma membranes of red blood cells are exceptionally elastic, an important feature which allows them to pass through capillaries narrower than their own diameter [65]. During the aging process, RBCs’ plasma membranes lose their elasticity, which results in a holdback of the RBCs at the inter-endothelial silts of the red pulp. This enables the macrophages located in the cords of the red pulp to phagocytize the RBCs that are too rigid to pass [66]. This erythrocyte rigidity has been shown to be crucial for α_v_-integrin-mediated erythrophagocytosis [67]. In addition, it has been demonstrated that the splenic environment plays a crucial role in facilitating erythrocyte turnover by inducing hemolysis [64]. Aged RBCs express a variety of adhesion molecules that interact with the extracellular matrix within the spleen. This adhesion molecule- driven restraining is important for the shrinkage of the cell, which has been demonstrated to result in hemolysis [64]. Interestingly, Klei et al., propose that iron recycling of heme from the extracellular splenic environment may be more efficient than recycling iron from the phagolysosome [64]. It was shown that senescent RBCs undergo hemolysis in the spleen releasing hemoglobin to the environment, which may bind to CD163, the hemoglobin haptoglobin scavenger receptor, which is highly expressed in the spleen [68]. This highlights the important role of the splenic environment for efficient iron recycling from senescent RBCs [64].

**Figure 5:**
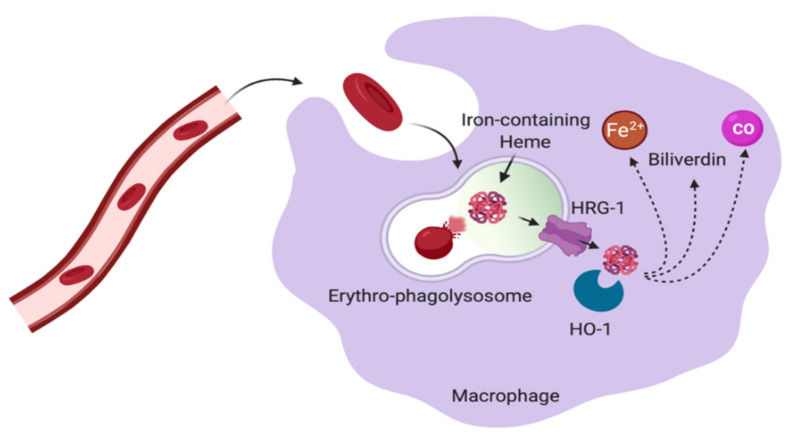
**Erythrophagocytosis by macrophages.** Macrophages in the red pulp of the spleen destroy senescent red blood cells and recycle the stored iron to be further incorporated into maturating RBCs during erythropoiesis. Senescent RBCs are engulfed into the phagolysosome of macrophages. Within the erythro-phagolysosome, RBCs are digested resulting in the breakdown of hemoglobin. Iron-containing heme is transported into the cytosol via HRG1. In the cytosol, iron bound to heme is processed by HO-1, resulting in the release of Fe^2+^, biliverdin and CO. The illustration was created using BioRender.com, (accessed on 3 April 2021).

When RBCs are internalized into the phagosome of macrophages, the phagosome merges with lysosomal vesicles to form phagolysosomes. In the phagolysosome, RBCs are digested resulting in the breakdown of hemoglobin. Further, heme-bound iron is then transported from the erythro-phagolysosomes into to the cytosol via a mechanism assisted by the heme responsive gene-1 (HRG1) transporter [1,69]. As soon as heme is present in the cytosol of macrophages, iron is processed from heme by heme oxygenase 1 (HO-1), whose expression is induced after heme accumulation in the cytoplasm [1,70] (Figure 5). It has been shown that HO-1 is crucial for survival and function of iron-metabolizing macrophages, as HO-1 deficiency caused a depletion of red pulp macrophages and bone marrow macrophages [71,72]. HO-1 breaks down heme-iron into Fe^2+^ and two heme degradation products, namely, biliverdin, bilirubin and also carbon monoxide [50].

### 5.3. Liver Kupffer Cells

Kupffer cells, the liver macrophage population, also express complex machinery for red blood cell clearance and heme iron recycling [19]. Liver Kupffer cells (KCs) are yolk sac derived or arise from fetal hematopoietic stem cells and reside within the liver sinusoids [50]. Today, it is accepted that KCs derive from colony-stimulating factor 1 receptor positive erythromyeloid progenitors from the yolk sac that migrate to the liver around embryonic day (E) 10.5 in mice [73]. Liver Kupffer cells are predominantly identified as CD45^+^F4/80^+^CD11b^intermediate^ cells [73]. As many iron-handling mechanisms and functions in the liver are managed by KCs, it is estimated that these nonmigratory KCs constitute the largest tissue-resident macrophage pool in the body [74]. KCs express iron regulating genes and with their residence within the liver sinusoid and non-migratory behavior, these liver macrophages are thought to be the primary cells to take up excessive iron to dampen hepatocyte overload [75]. Furthermore, liver KCs can have an inhibitory effect on liver hepcidin expression independent of inflammation, implicating the ability of KCs to bidirectionally regulate liver hepatic iron content in an inflammation-dependent process [76].

Iron can be taken up in various forms such as, hemoglobin-bound iron, transferrin-bound iron or free iron, by macrophages through CD163, TfR1 and DMT1 respectively [48]. Iron produced by heme catabolism from macrophages is either stored intracellularly bound to ferritin or exported through FPN, which is abundantly expressed on all iron-metabolizing macrophages [1]. Thus, macrophages are needed to maintain the steady-state levels of iron and prevent toxic iron accumulation in the body [1]. In addition to their dominant role in scavenging senescent red blood cells and maintaining the iron body level in balance, macrophages can produce hepcidin locally the site of infection to limit iron bioavailability for pathogens [1].

## 6. Monocytes and Their Contribution to Iron Metabolism

### 6.1. Monocyte Populations

Monocytes are bone marrow-derived, circulating leukocytes that are key players in tissue homeostasis [77]. Circulating blood monocytes are a heterogeneous cell population and constitute a crucial component of innate immunity [78]. Under steady-state conditions, circulating blood monocytes act as precursors to restore tissue-resident macrophages and dendritic cells [79]. This was already proposed in the 1960s by van Furth and Crohn, where they used [^3^H] thymidine labelling to determine the kinetics of monocyte migration in the blood circulation and their homing to tissues. Monocytes stayed a few days in the circulation and then migrated to various tissues [80]. Their data led to the conclusion that monocytes continuously replenish tissue-resident macrophages [80]. Today, two populations of monocytes have been described in mice [78]. These two populations are discriminated by different expressions of lymphocyte antigen 6C (Ly6C) (Table 1). Ly6C^high^-expressing monocytes have pro-inflammatory functions and express high levels of C-C chemokine receptor 2 (CCR2) [81]. These monocytes expressing high levels of Ly6C can transport antigens into the lymph node and accumulate at the site of inflammation, where they differentiate into macrophages or dendritic cells, depending on the local cytokine environment [82]. The second monocyte subset expresses low levels of the lymphocyte antigen C6, so-called LyC6^low^ monocytes [78]. This population is known for patrolling along the lumen of the vasculature, and they are essential for an early response in inflammation and tissue repair [78].

In humans, three functionally different monocyte subsets are known. In the peripheral blood, around 90% of the circulating monocytes express high levels of CD14 and no CD16 [77]. Further, the subset which expresses CD16 can be divided into intermediate and non-classical monocytes. The intermediate subpopulation has high expression levels of CD14, whereas the non-classical subtype shows low level of CD14 expression, accompanied by high levels of CD16 expression [77]. Cross species analysis revealed that the human CD14^+^CD16^−^ monocyte subset is the counterpart of the Ly6C^high^CCR2^high^ monocyte subtype in mice [83].

### 6.2. Monocytes and Iron Handling

Under normal physiological conditions, most extracellular iron is tightly bound to Tf [84]. Physiologically and also to prevent toxic iron overload, circulating iron is taken up via TfR1 and stored in stable ferritin complex, primarily in hepatocytes [25]. Circulating iron not bound to transferrin, heme or ferritin (so-called NTBI), is in contrast to transferrin-bound iron, toxic. By the formation of reactive oxygen species, this non-transferrin bound iron causes cellular damage [85]. For a long time, monocytes were thought to contribute directly to the iron turnover as progenitors of macrophages [86]. Like macrophages, monocytes express erythrocyte-scavenging receptors, and are thus able to phagocytose damaged red blood cells [72,73]. Recently, a study on human myelomonocytic cells showed that the classical (CD14^++^CD16^−^) and intermediate (CD14^+^CD16^+^) human monocytes express iron handling proteins, such as FPN, DMT 1 and TfR1 and can take up NTBI and keep the iron in ferritin-bound form [84]. Additionally, these classical and intermediate monocytes showed efficient erythrocyte phagocytosis capacity [84]. Consequently, monocytes could have a standalone role in iron metabolism, contributing to iron homeostasis and protecting the organism from toxic iron accumulations [84].

## 7. Iron, Immunity and Infection

The immune system is a complex network with specialized cells to protect the host from pathogenic organisms and infections [39]. Iron not only is an essential element for the human body, it is also an important cofactor in basic metabolic processes in pathogenic microorganisms [87]. Iron is crucial for microbial growth [39]. Changes in iron availability and distribution within the host result in significant effects on the pathogen virulence [18]. As pathogens invade the organism, they thrive on free iron in the circulation to proliferate and advance their attack [14]. An iron-based resistance mechanism against extracellular pathogens evolved by reducing the circulating iron in the system [88]. This iron sequestration is mediated by the increased expression of IL-1 and IL-6 of immune cells, which enhances hepcidin synthesis and blocks intracellular iron efflux [89]. By inhibiting the cellular iron efflux, innate immunity deprives the pathogen of the accessible iron and arrests its growth and dissemination [89,90]. This response of decreasing plasma iron concentration within hours of infection is referred to as hypoferremia of inflammation [91].

It has been shown that there is an increased susceptibility to infections in individuals with increased plasma iron levels due to thalassemia or primary hemochromatosis [92]. In contrast, mild forms of iron deficiency can be protective against *Plasmodium falciparum*, causing malaria infection [93]. Nevertheless, certain intracellular microbes evolved specialized techniques to sequester the metal from the environment to ensure their own survival [14]. For example, a wide range of Gram-positive and Gram-negative bacteria can access iron with the help of siderophores [94], low molecular weight high-affinity iron-binding complexes, which compete with Tf for iron sequestration [82,83]. Siderophores are synthesized by bacteria if the iron availability is low in the host. The siderophores are then secreted into the extracellular environment where they bind ferric iron with high affinity and ease its use for the pathogen [15]. Besides siderophores, many pathogens have evolved heme uptake systems as most of the iron found in the human body is bound to hemoglobin. Until today, two main classes of bacterial heme acquisition systems are known. First, in Gram-negative bacteria, heme can be directly taken up by the pathogen [95]. Heme uptake systems bind heme-containing proteins at their outer membrane and transport heme into the periplasm in a TonB dependent manner [95]. Second the uptake of hemoglobin can be hemophore-dependent [95]. Hemophores are proteins secreted from the bacterial cell, binding heme and transporting it to a receptor on the cell surface [96]. Whether the uptake is hemophore-dependent or not, both acquisition systems contain cell surface receptors, which bind heme and complex machinery then shuttle the heme across the cell wall, and cytoplasmic components then release iron from the heme for the pathogen’s use [15].

The human immune system counteracts these mechanisms by withholding iron from the circulation to prevent the pathogens proliferation, displaying the precious role of iron for immunity and infection, for host and pathogen [15].

## 8. Conclusions

Iron homeostasis has a complex regulation. As iron is toxic when present in excess, iron availability is tightly controlled at the cellular and the systemic level. Roughly 25 mg of iron per day is required for erythropoiesis. Dietary iron supply (1–2 mg) is not sufficient to meet the daily iron needs for erythropoiesis. Therefore, macrophages in the liver, spleen and bone marrow recycle iron from senescent red blood cells, which are then re-used for erythropoiesis. If tight regulation of iron availability is lost, it can lead to severe cellular damage and systemic disease.

## Figures and Tables

**Table 1:** **Mouse and human monocyte subsets**. Mouse and human monocyte subsets enlisted with their primary markers and function according to Sprangers et al. [81].

Subset	Marker	Function
Mouse		
Classical	Ly6C^high^CD11b^+^	Proinflammatory
Non-classical	Ly6C^low^CD11b^+^	Patrolling
Human Classical	CD15^+^CD16^−^	Immune response
Intermediate	CD14^+^CD16^+^	Proinflammatory
Non-classical	CD14^+^CD16^++^	Patrolling

## Data Availability

Not applicable.

## Abbreviations

BM	Bone marrow
DMT1	Divalent metal transporter 1
FPN	Ferroportin
Ft	Ferritin
Hgb	Hemoglobin
HO-1	Heme oxygenase
IFNγ	Interferon-gamma
IL-1	Interleukin-1
IL-6	Interleukin 6
IRE	Iron responsive elements
IRP1	Iron regulating protein 1
IRP2	iron regulating protein 2
KC’s	Kupffer cells
MΦ	Macrophage
NTBI	Non transferrin-bound iron
RBC	Red blood cell
RPM	Red pulp macrophages
TBI	Transferrin-bound iron
Tf	Transferrin
TfR1	Transferrin receptor 1
TfR2	Transferrin receptor 2
